# The relationship between insulin resistance, serum alkaline phosphatase, and left ventricular dysfunction following myocardial infarction

**DOI:** 10.1038/s41598-023-45246-5

**Published:** 2023-10-20

**Authors:** Qifeng Guo, Mengdan Miao, Linan Duan, Yongsheng Liu, Yahui Qiu, Xuejuan Feng, Shisen Liang, Weiqiang Xiao, Mingqi Zheng, Mei Wei, Gang Liu

**Affiliations:** 1 Department of Heart Center, the First Hospital of Hebei Medicical University, 89Donggang Road, Shijiazhuang, 050000 Hebei China; 2 Graduate School of Hebei Medical University, 361 Zhongshan East Road, Shijiazhuang, 050000 Hebei China; 3 Hebei Key Laboratory of Heart and Metabolism, Shijiazhuang, 050000 Hebei China; 4 Department of Geriatric Medicine, the First Hospital of Hebei Medicical University, 89 Donggang Road, Shijiazhuang, 050000 Hebei China

**Keywords:** Cardiology, Diseases, Endocrinology, Medical research

## Abstract

The occurrence of heart failure following acute myocardial infarction (AMI) significantly increases the risk of post-infarction mortality. Alkaline phosphatase (AP) is considered to be an independent predictor of cardiovascular disease (CVD) and adverse outcomes. Furthermore, in recent years, alkaline phosphatase has been associated with insulin resistance (IR). Our aim was to investigate the correlation between IR substitutes (triglyceride-glucose (TyG) index, triglyceride to high-density lipoprotein cholesterol (TG/HDL-C) ratio), AP, and LV dysfunction in patients admitted after AMI. The retrospective study included 810 patients who underwent coronary angiography for myocardial infarction at the First Hospital of Hebei Medical University from August 2018 to December 2021. Patients were categorized into three groups based on their serum AP levels. Clinical characteristics at admission, cardiac echocardiography findings, coronary angiography results, and biochemical markers such as serum AP levels and triglycerides (TG) were recorded during hospitalization. Left ventricular ejection fraction (LVEF) was assessed using cardiac echocardiography conducted from the time of admission until the coronary angiography procedure. A total of 774 patients with AMI were included in this study. The TyG index is significantly correlated with the TG/HDL-C ratio. (R = 0.739, *P* < 0.001). Binary logistic regression analysis revealed that elevated serum AP (OR 2.598, 95% CI 1.331–5.071, *P* = 0.005), presence of the left anterior descending (LAD) artery as the infarct-related artery (IRA) (OR 2.452, 95% CI 1.352–4.449, *P* = 0.003), and triglyceride (TG) levels (OR 0.652, 95% CI 0.429–0.992, *P* = 0.046) were protective risk factor for an admission LVEF < 40% following AMI. The serum alkaline phosphatase and LAD as IRA are independent risk factors for severe reduction in LVEF during hospitalization for AMI. Conversely, triglyceride are independent protective factor for severe reduction in LVEF during AMI hospitalization.

## Introduction

The incidence of myocardial infarction (MI) has been increasing over the years, attracting significant attention. Although the mortality rate from AMI has decreased with the advent of percutaneous coronary intervention (PCI), myocardial cell loss resulting from heart attacks remains a major cause of heart failure. Heart failure significantly increases the risk of mortality after a myocardial infarction. Patients with LVEF < 40% are defined as having severely reduced LVEF, and compared to patients with LVEF ≥ 40%, those with LVEF < 40% exhibit a significantly lower one-year survival rate^1,2^. In search of prognostic biomarkers, IR surrogate markers and serum AP levels have garnered considerable interest among researchers.

Insulin resistance is a hallmark of metabolic syndrome (MetS), and high IR levels not only increase the risk of CAD but also significantly correlate with adverse cardiovascular outcomes^3,4^. However, direct measurements of IR (such as hyperinsulinemic-euglycemic clamp and insulin suppression test) are invasive, costly, and complex procedures^5^. Epidemiological studies require simple and easily obtainable markers of IR. In this context, the TyG index and TG/HDL-C ratio have been confirmed by studies as surrogate markers of IR^6,7^. The TyG index, composed of TG and FBG (fasting blood glucose), is calculated using the formula: TyG = ln[fasting triglycerides (mg/dl) × fasting blood glucose (mg/dl)/2]^8^.

Furthermore, alternative markers of IR are associated with poor prognosis in patients with acute ST-segment elevation myocardial infarction (STEMI) and non-ST elevation acute coronary syndrome (NSTE-ACS) after percutaneous coronary intervention (PCI) treatment^9,10^. In recent years, Professor Da-Hye Son discovered Serum AP levels are independently and positively associated with surrogate markers of insulin resistance in Korean adults^11^. Elevated serum AP levels not only contribute to vascular calcification^12^ but are also associated with adverse prognosis in CAD^13^. Therefore, our aim was to investigate the relationship between IR surrogate markers (TyG index and TG/HDL-C ratio) and serum AP with severe reduction in LVEF at admission in a population of first-time AMI patients, providing insights for predicting adverse cardiovascular events.

## Materials and methods

### Study population

The study is a retrospective study that selected a total of 810 patients who underwent coronary angiography due to AMI at the First Hospital of Hebei Medical University from August 2018 to December 2021 as the study subjects. The inclusion criteria were as follows: (1) AMI (2) undergoing coronary angiography. The exclusion criteria were as follows: (1) previous coronary intervention, thrombolytic therapy, or coronary artery bypass grafting; (2) history of heart failure, cardiomyopathy, congenital heart disease, or valvular heart disease; (3) chronic dialysis; (4) chronic liver disease, active hepatitis, or severe liver dysfunction; (5) severe acute infection; (6) acute or chronic hepatobiliary diseases, including chronic inflammatory diseases involving the skeletal system; (7) malignancy; (8) suspected familial hypertriglyceridemia [plasma triglycerides (TG) ≥ 500 mg/dL (5.65 mmol/L) or having a first-degree relative with TG ≥ 500 mg/dL]; (9) incomplete clinical data. The study was performed according to the guidelines of the Helsinki Declaration and was approved by the Ethics Committee of The First Hospital of Hebei Medical University (20,200,511).

### Data collection and definitions

Clinical data were collected by trained clinical doctors from medical records, including medical history, baseline clinical and demographic characteristics. The records indicated that all patients had blood biochemical parameters, complete blood count, and cardiac echocardiography collected upon admission. All blood samples were collected from the antecubital vein after a 12-h overnight fasting following admission. Left ventricular ejection fraction was obtained through cardiac echocardiography at admission and before coronary angiography. Systolic and diastolic blood pressures were measured in the right arm of the patients using a standard mercury sphygmomanometer after the patients had been seated and rested for 10 min. The triglyceride-glucose index was defined as TyG = Ln[fasting triglycerides (mg/dL) × fasting glucose (mg/dL)/2]^8^. The triglyceride to high-density lipoprotein cholesterol ratio was calculated as TG (mg/dL) divided by HDL-C (mg/dL). Diabetes mellitus was defined as fasting blood glucose (FBG) ≥ 7.0 mmol/L, 2-h postprandial blood glucose (PBG) ≥ 11.1 mmol/L, glycated hemoglobin (HbA1c) ≥ 6.5%, or use of any hypoglycemic medications or self-reported history of diabetes. Hypertension was defined as systolic blood pressure (SBP) ≥ 140 mmHg or diastolic blood pressure (DBP) ≥ 90 mmHg, use of any antihypertensive medications, or self-reported history of hypertension. Dyslipidemia was defined as any self-reported history or use of lipid-lowering medications or total cholesterol (TC) ≥ 5.17 mmol/L. Left ventricular systolic dysfunction was defined as LVEF < 40%. Atrial fibrillation (AF) was defined as AF on admission electrocardiogram or a history of AF. Ischemic time was defined as the time from the onset of typical AMI symptoms to admission. The time for revascularization is defined as the duration from hospital admission to the performance of PCI or CABG. In this study, acute myocardial infarction included STEMI and non-ST-elevation myocardial infarction (NSTEMI), and was in accordance with the guidelines of the European Society of Cardiology^14,15^.

### Angiographic analysis

The angiographic data were retrieved from the records of the cardiac catheterization laboratory. Coronary angiography procedures were conducted by three specialized interventionists, either through the radial or femoral route, based on the operator's discretion. To ensure consistency and minimize inter-observer variability, the assessment of coronary arteries was performed by three senior cardiologists. If the degree of coronary artery stenosis is equal to or greater than 50%, we consider the presence of significant CAD. The complexity of coronary atherosclerosis was quantified using the Gensini score, which takes into account both angiographically significant and nonsignificant stenosis. The Gensini score for each patient was determined by summing up the scores of individual coronary arteries. To facilitate the calculation of the Gensini score, a minimum of five different views were obtained for each patient. The degree of stenosis and the coronary artery lesion site were scored as follows: 1 point for ≤ 25% narrowing, 2 points for 26–50% narrowing, 4 points for 51–75% narrowing, 8 points for 76–90% narrowing, 16 points for 91–99% narrowing, and 32 points for total occlusion. Thereafter, multiply the score assigned to each lesion by the relevant coefficient based on the location and significance of the lesion within the coronary artery (5 for the left main coronary artery, 2.5 for the proximal segment of the left anterior descending coronary artery, 2.5 for the proximal segment of the circumflex artery, 1.5 for the mid-segment of the left anterior descending coronary artery, 1.0 for the right coronary artery, the distal segment of the left anterior descending coronary artery, the posterolateral artery, and the obtuse marginal artery, and 0.5 for other segments). Finally, add up the obtained scores to calculate the Gensini score^16^.

### Statistical analysis

Serum AP tertile were categorized as follows: Low: < 66 U/L, Middle: 67–85 U/L, High: > 85U/L. Continuous variables were presented as mean ± standard deviation for normally distributed variables and as median (interquartile range) for non-normally distributed variables. Student's t-test was employed to compare the normally distributed values between different serum AP levels groups. Mann–Whitney U test was used to assess differences in non-normally distributed values among different serum AP levels groups. The correlation between the TyG index and other parameters was assessed using the Spearman rank correlation test.The χ^2^ test or Fisher exact test was used for categorical variables, as appropriate. Univariate regression analysis was performed to evaluate the association between clinical variables and severe reduction in LVEF. Variables with *P* < 0.05 on univariate analysis were considered as confounding factors and entered the multivariate regression analysis. The calibration was assessed using the Hosmer–Lemeshow goodness-of-fit test. A two-sided analysis with a *P* value < 0.05 was considered statistically significant. All statistical analyses were performed using SPSS version 26.0.

### Ethics approval and consent to participate

The study was performed according to the guidelines of the Helsinki Declaration and has been approved by the ethics committees at the First Hospital of Hebei Medical University, China. Since data were evaluated retrospectively, pseudonymously and were solely obtained for treatment purposes, a requirement of informed consent was waived by the Ethics Committee of the First Hospital of Hebei Medical University.

## Results

### Baseline characteristics of patients in different serum AP levels groups

**Figure 1:**
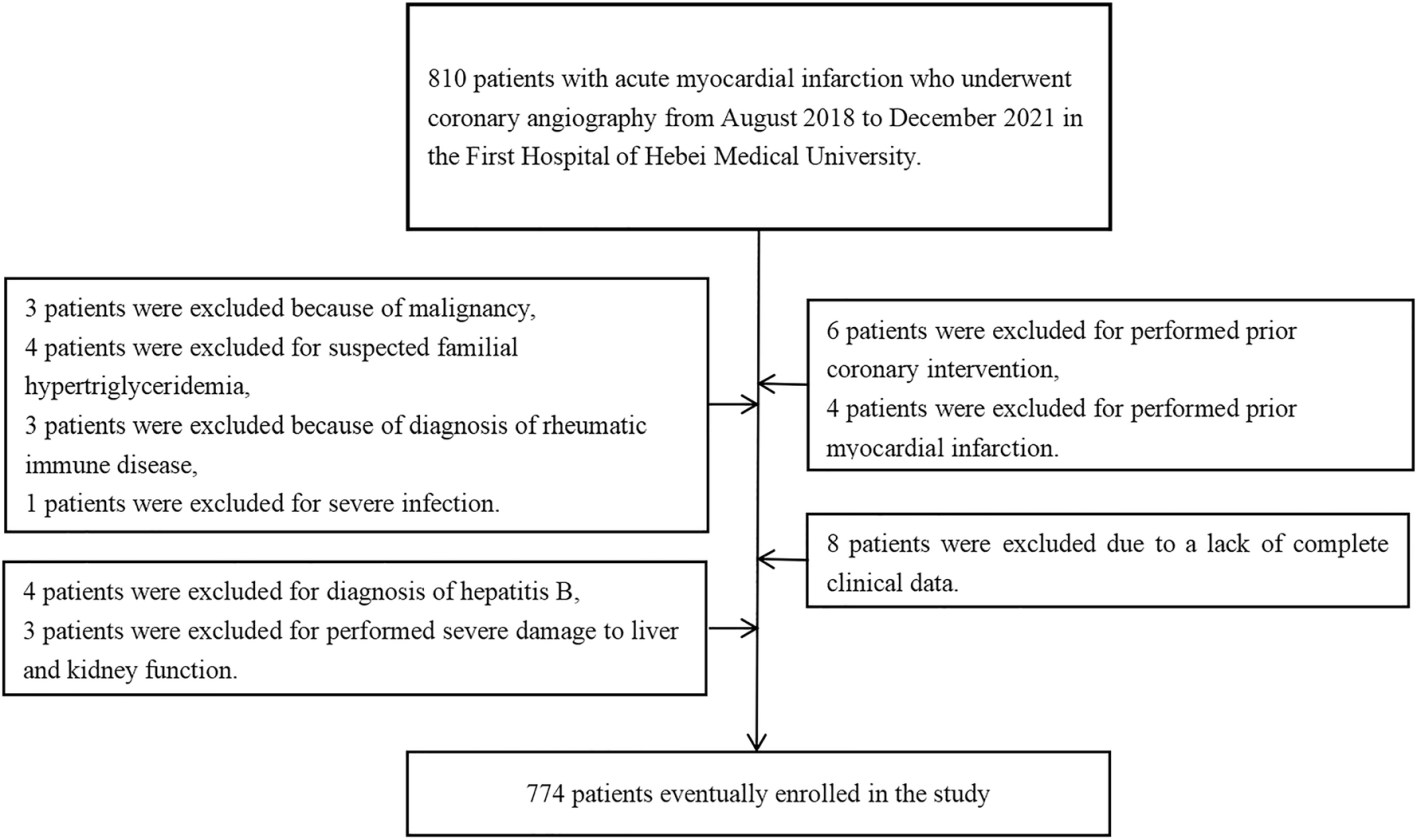
Flow chart.

**Figure 2:**
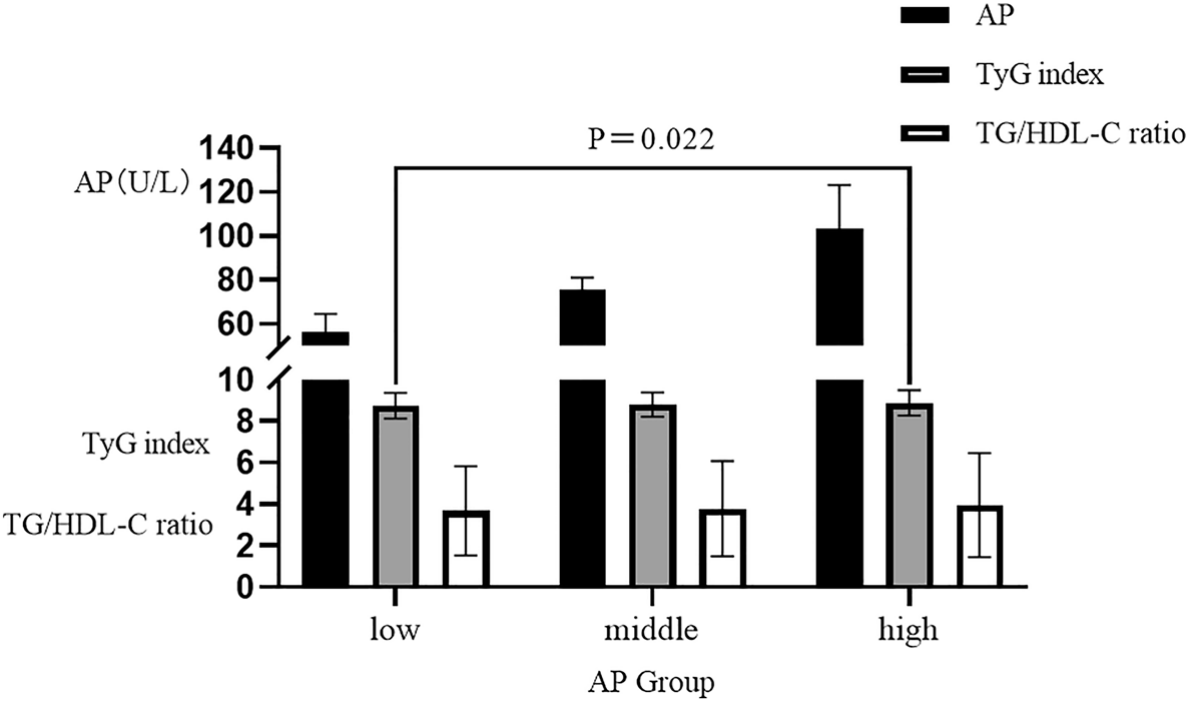
Relationship between serum alkaline phosphatase and TyG index and TG/HLD-C ration. *AP* alkaline phosphatase; *TyG* Triglyceride-glucose; *TG/HDL-C ratio* Triglyceride/high-density lipoprotein cholesterol ratio.

The final study population consisted of 774 patients who underwent coronary angiography for AMI (Fig. 1). All patients were divided into three groups based on their serum AP levels. The baseline characteristics of the three groups are presented in Table 1, the significant differences were observed among the groups in terms of ischemic time, dyslipidemia, LVEF < 40%, white blood cell count (WBC), low-density lipoprotein cholesterol (LDL-C), total cholesterol (TC), Apolipoprotein B (ApoB), and TyG index significantly increased with increasing tertiles, while it significantly decreases with the increase in tertiles for diabetic patients (*P* < 0.05). No statistically significant differences were found for other variables (Table 1). Regarding the TyG index, a significant difference was observed only between the high serum AP levels group and the low serum AP levels group (*P* = 0.022). No significant differences in the TG/HDL-C ratio were observed among the various groups categorized by serum AP (Fig. 2).

**Table 1:** Comparison of baseline characteristics of patients in different serum AP levels groups.

Items	Total population	COV	Serum AP level Tertile						*P* value
			Low	COV	Middle	COV	High	COV	
n	774		254		263		257		
Clinical characteristics									
Age, years	62.00 (52.00–69.00)	0.18	63.00 (54.00 − 70.00)	0.17	62.00 (51.00–70.00)	0.19	61.00 (51.50 − 67.00)	0.19	0.090
Male, n (%)	597 (77.13)	–	208 (81.89)	–	197 (74.91)	–	192 (74.71)	–	0.088
Ischemic time, hour	18.00 (6.00–72.00)	41.39	17.00 (6.00 − 72.00)	37.00	12.00 (5.00–48.00)	89.68	24.00 (6.50 − 96.00)	13.22	0.042
Time for revascularization, hour	77.00 (24.00–158.00)	1.34	71.00 (22.00–156.00)	1.49	79.00 (23.25.00–156.00)	1.20	89.00 (27.75 − 163.00)	1.21	0.862
Diabetes mellitus, n (%)	196 (25.32)	–	79 (31.10)	–	60 (22.81)	–	57 (22.18)	–	0.035
Hypertension, n (%)	452 (58.40)	–	154 (60.63)	–	149 (56.65)	–	149 (57.98)	–	0.648
Dyslipidemia, n (%)	207 (26.74)	–	51 (20.08)	–	72 (27.38)	–	84 (32.68)	–	0.005
Current Smoking, n (%)	253 (32.69)	–	77 (30.31)	–	89 (33.84)	–	87 (33.85)	–	0.617
Family History of CAD, n (%)	31 (4.01)	–	6 (2.36)	–	15 (5.70)	–	10 (3.89)	–	0.152
Family History of Cerebrovascular disease , n (%)	125 (16.15)	–	41 (16.14)	–	38 (14.45)	–	46 (17.90)	–	0.565
Atrial fibrillation, n (%)	28 (3.62)	–	10 (3.94)	–	14 (5.32)	–	4 (1.56)	–	0.067
STEMI, n (%)	399 (51.55)	–	120 (47.24)	–	134 (50.95)	–	145 (56.42)	–	0.113
Vital signs at admission									
SBP, mmHg	129.00 (115.00 − 143.25)	0.17	127.00 (113.00–144.00)	0.18	132.00 (116.00 − 143.00)	0.15	128.00 (115.00 − 144.00)	0.18	0.530
DBP, mmHg	80.00 (72.00 − 89.00)	0.17	79.00 (72.00–89.00)	0.16	80.00 (73.00 − 89.00)	0.16	80.00 (72.00 − 90.00)	0.18	0.396
RHR, beats per minute	75.00 (66.00 − 85.00)	0.22	74.50 (65.00–82.00)	0.21	75.00 (67.00 − 85.00)	0.22	76.00 (67.00 − 88.00)	0.22	0.068
Laboratory characteristics									
WBC , 10^9/L	8.40 (6.60 − 10.60)	0.41	7.80 (6.30–10.10)	0.45	8.60 (6.60 − 10.50)	0.36	9.00 (6.80 − 11.60)	0.40	0.001
Hb , g/L	138.00 (126.00 − 148.00)	0.13	139.00 (125.75–146.00)	0.13	137.00 (126.00 − 148.00)	0.14	139.00 (127.50 − 150.00)	0.13	0.311
FBG, mmol/L	5.61 (4.86 − 7.27)	0.62	5.56 (4.78–7.10)	0.68	5.61 (4.91 − 6.92)	0.56	5.63 (4.94 − 7.84)	0.61	0.247
Creatinine, μmol/L	70.50 (61.68 − 81.30)	0.31	71.25 (63.00–83.30)	0.29	70.90 (61.60 − 81.00)	0.33	68.60 (60.20 − 79.25)	0.30	0.096
BUN, mmol/L	5.28 (4.24 − 6.53)	0.48	5.43 (4.48–6.61)	0.41	5.24 (4.23 − 6.56)	0.56	5.06 (4.10 − 6.48)	0.46	0.074
LDL–C, mmol/L	2.90 (2.38 − 3.36)	0.26	2.75 (2.35–3.31)	0.25	2.92 (2.35 − 3.39)	0.25	2.99 (2.47 − 3.43)	0.27	0.032
HDL–C, mmol/L	0.97 (0.84 − 1.11)	0.27	0.96 (0.83–1.07)	0.20	0.96 (0.84 − 1.11)	0.26	0.99 (0.84 − 1.16)	0.33	0.116
TG, mmol/L	1.32 (0.98 − 1.86)	0.64	1.27 (0.91–1.81)	0.63	1.30 (1.05 − 1.86)	0.64	1.39 (1.07 − 1.90)	0.63	0.096
TC, mmol/L	4.59 (3.88 − 5.22)	0.23	4.46 (3.82–5.01)	0.21	4.59 (3.86 − 5.21)	0.22	4.78 (4.00 − 5.38)	0.24	0.006
UA, μmol/L	331.60 (279.65 − 402.05)	0.38	340.50 (287.40–413.48)	0.29	331.50 (286.1 − 397.00)	0.32	322.60 (272.00 − 400.05)	0.51	0.249
Lp(a), mg/L	212.30 (104.80 − 417.00)	1.54	214.40 (110.40–440.90)	1.57	215.05 (109.75 − 415.45)	1.56	206.40 (94.18 − 400.42)	1.49	0.335
ApoA1, g/L	1.12 (1.00 − 1.24)	0.19	1.11 (1.00–1.21)	0.17	1.10 (0.98 − 1.24)	0.20	1.14 (1.01 − 1.26)	0.20	0.222
ApoB, g/L	0.82 (0.66 − 0.97)	0.29	0.81 (0.64–0.94)	0.28	0.81 (0.67 − 0.96)	0.29	0.86 (0.69 − 1.01)	0.28	0.013
AP, U/L	75.50 (63.00 − 90.25)	0.31	58.00 (52.00–63.00)	0.14	75.00 (71.00 − 80.00)	0.07	98.00 (90.50 − 109.00)	0.21	< 0.001
TyG index	8.74 (8.39 − 9.20)	0.07	8.67 (8.31–9.15)	0.07	8.82 (8.39 − 9.16)	0.07	8.79 (8.45 − 9.29)	0.07	0.027
TG/HDL ratio	3.23 (2.22 − 4.63)	0.74	3.15 (2.06–4.64)	0.71	3.19 (2.27 − 4.60)	0.74	3.31 (2.26 − 4.69)	0.78	0.597
Infarct related artery, n (%)									
LM ,n (%)	7 (0.90)	–	4 (1.57)	–	1 (0.38)	–	2 (0.78)	–	0.529
LAD, n (%)	446 (57.62)	–	138 (54.33)	–	150 (57.03)	–	158 (61.48)	–	0.255
LCX, n (%)	138 (17.83)	–	54 (21.26)	–	44 (16.73)	–	40 (15.56)	–	0.206
RCA, n (%)	183 (23.64)	–	58 (22.83)	–	68 (25.86)	–	57 (22.18)	–	0.574
Extent of coronary artery disease,n (%)									
1–vessel, n (%)	183 (23.64)	–	52 (20.47)	–	69 (26.24)	–	62 (24.12)	–	0.382
2–vessel, n (%)	238 (30.75)	–	75 (29.53)	–	78 (29.66)	–	85 (33.07)	–	
3–vessel, n (%)	353 (45.61)	–	127 (50.00)	–	116 (44.11)	–	110 (42.80)	–	
Gensini score	56.00 (38.00–88.00)	0.77	62.00 (40.00–92.00)	0.73	52.00 (36.00–84.00)	0.80	57.50 (35.50–92.00)	0.74	0.144
Echocardiography									
LVEF, %	56.00 (46.00–63.00)	0.20	57.00 (46.75–63.00)	0.20	56.00 (46.00–63.00)	0.20	53.00 (45.00–63.00)	0.22	0.179
LVEF < 40%, n (%)	78 (10.08)	–	17 (6.69)	–	26 (9.98)	–	35 (13.62)	–	0.034
Prior medication									
Antiplatelets, n (%)	23 (2.97)	–	8 (3.15)	–	5 (1.90)	–	10 (3.89)	–	0.401
Beta–blocker, n (%)	25 (3.23)	–	11(4.33)	–	8 (3.04)	–	6 (2.34)	–	0.433
Calcium inhibitor, n (%)	177(22.87)	–	61(24.02)	–	56 (21.29)	–	60 (23.35)	–	0.743
ACEI, n (%)	28 (36.18)	–	12 (4.72)	–	12 (4.56)	–	4 (1.56)	–	0.095
Angiotensin II antagonist, n (%)	40 (5.16)	–	16 (6.30)	–	13 (4.94)	–	11 (4.28)	–	0.576
Diuretic, n (%)	10 (1.29)	–	5 (1.97)	–	3 (1.14)	–	2 (0.78)	–	0.448
Insulin, n (%)	37 (4.78)	–	15 (5.91)	–	13 (4.94)	–	9 (3.50)	–	0.439
Oral antidiabetic drug, n (%)	91 (11.76)	–	38 (14.96)	–	23 (8.75)	–	30 (11.67)	–	0.090
Statin, n (%)	22 (2.84)	–	11 (4.33)	–	4 (1.52)	–	7 (2.72)	–	0.156
Comorbidities									
Atrial fibrillation, n (%)	28 (3.62)	–	10 (3.94)	–	14 (5.32)	–	4 (1.56)	–	0.067
Ventricular tachycardia, n (%)	9 (1.16)	–	3 (1.18)	–	4 (1.52)	–	2 (0.78)	–	0.847
Heart arrest, n (%)	8 (1.03)	–	3 (1.18)	–	4 (1.52)	–	1 (0.39)	–	0.504
Pulmonary infection, n (%)	37 (4.78)	–	9 (3.54)	–	14 (5.32)	–	14 (5.45)	–	0.528
Hydropericardium, n (%)	6 (0.78)	–	1 (0.39)	–	2 (0.76)	–	3 (1.27)	–	0.706
Type of revascularization, n (%)									
PCI, n (%)	621 (80.23)	–	201 (79.13)	–	221 (84.03)	–	199 (77.43)	–	0.145
CABG, n (%)	28 (3.62)	–	12 (4.72)	–	8 (3.04)	–	8 (3.11)	–	0.514

*TyG* triglyceride-glucose, *TG/HDL-C ratio* triglyceride/high-density lipoprotein cholesterol ratio, *COV* coefficient of variation, *STEMI* ST-elevation myocardial infarction, *WBC* white blood cell, *ACEI* angiotensin converting enzyme inhibitor, *Hb* hemoglobin, *CAD* coronary artery disease, *SBP* systolic blood pressure, *DBP* diastolic blood pressure, *RHR* resting heart rate, *LM* left main coronary artery, *LAD* left anterior descending artery, *LCX* left circumflex artery, *RCA* right coronary artery, *FBG* fasting blood glucose, *LDL-C* low-density lipoprotein cholesterol, *HDL-C* high-density lipoprotein cholesterol, *TG* triglycerides, *TC* total cholesterol, *UA* uric acid, *Lp(a)* lipoprotein (a), *ApoA1* apolipoprotein A1 ApoB apolipoprotein B, *BUN* blood urea nitrogen, *LVEF* left ventricular ejection fraction, *AP* alkaline phosphatase, *PCI* percutaneous coronary intervention, *CABG* coronary artery bypass grafting.

Dates are presented as mean ± SD, medians with inter quartile ranges or percentage.

### Correlation between the TyG index and clincal variables

Spearman's correlation analysis revealed that there was no significant correlation between the TyG index and serum AP groups, HDL-C, LVEF, and Gensini score. Weak relationships were observed between the TyG index and TC, as well as LDL-C. However, a strong correlation was found between the TyG index and the TG/HDL-C ratio (R = 0.739, *P* < 0.001) (Table 2).

**Table 2:** Correlation between the TyG index and clincal variables.

Parameters	R (Spearman)	*P* value
TG/HDL ratio	0.739	< 0.001
AP	0.120	0.001
TC	0.355	< 0.001
LDL-C	0.322	< 0.001
HDL-C	− 0.112	0.002
LVEF	− 0.077	0.032
Gensini score	0.097	0.007

*TyG* triglyceride-glucose, *TG/HDL-C ratio* triglyceride/high-density lipoprotein cholesterol ratio, *LDL-C* low-density lipoprotein cholesterol, *HDL-C* high-density lipoprotein cholesterol, *TC* total cholesterol, *LVEF* left ventricular ejection fraction, *AP* alkaline phosphatase.

### Evaluation of factors associated with LVEF < 40%

In the fully adjusted model, the serum AP level in the high group (OR 2.598, 95% CI 1.331–5.071, *P* = 0.005), LAD as IRA (OR 2.452, 95% CI 1.352–4.449, *P* = 0.003). Conversely, triglyceride (OR 0.652, 95% CI 0.429–0.992, *P* = 0.046) are independent protective factors for severe reduction in LVEF during AMI hospitalization (Table 3).

**Table 3:** Univariate and multivariate analysis of TyG index and LVEF < 40%.

Variable	Univariate analysis		Multivariate analysis	
	OR (95%CI)	*P* value	OR (95%CI)	*P* value
Age	1.026 (1.004–1.049)	0.020	1.012 (0.986–1.038)	0.376
Male	1.371 (0.812–2.317)	0.238		
SBP	0.979 (0.968–0.991)	0.001	0.983 (0.970–0.996)	0.012
DBP	0.985 (0.967–1.003)	0.104		
RHR	1.020 (1.007–1.034)	0.002	1.014 (1.000–1.029)	0.055
Ischemic time	1.000 (0.999–1.003)	0.734		
WBC	1.014 (0.982–1.046)	0.401		
Hb	0.984 (0.973–0.995)	0.005	0.992 (0.979–1.005)	0.248
TyG	1.019 (0.695–1.494)	0.924		
FBG	1.084 (1.026–1.146)	0.004	1.016 (0.937–1.102)	0.702
TG	0.598 (0.410–0.873)	0.008	0.652 (0.429–0.992)	0.046
BUN	1.240 (1.144–1.343)	< 0.001	1.127 (0.996–1.275)	0.058
Creatinine	1.018 (1.009–1.027)	< 0.001	1.002 (0.989–1.015)	0.790
LDL-C	1.018 (0.744–1.392)	0.911		
HDL-C	1.765 (0.844–3.694)	0.131		
TC	1.050 (0.840–1.313)	0.666		
UA	1.002 (1.000–1.004)	0.012	1.000 (0.999–1.002)	0.651
ApoA1	1.597 (0.541–4.715)	0.396		
ApoB	1.594 (0.608–4.181)	0.343		
Lp(a)	1.000 (0.999–1.001)	0.954		
LAD as IRA	2.677 (1.548–4.628)	< 0.001	2.452 (1.352–4.449)	0.003
Extent of coronary artery disease				
1-vessel	1	–	1	–
2-vessel	1.94 (0.903–4.167)	0.089	1.545 (0.686–3.481)	0.294
3-vessel	2.463 (1.209–5.018)	0.013	1.266 (0.540–2.969)	0.587
Gensini score	1.012 (1.007–1.017)	< 0.001	1.009 (1.003–1.016)	0.003
Hypertension	0.814 (0.509–1.302)	0.390		
Diabetes mellitus	1.875 (1.147–3.063)	0.012	0.919 (0.399–2.119)	0.843
Dyslipidemia	1.617 (0.988–2.648)	0.056		
Current Smoking	1.326 (0.818–2.149)	0.253		
Atrial fibrillation	1.074 (0.317–3.640)	0.909		
Family history of CAD	1.339 (0.456–3.933)	0.595		
Family history of cerebrovascular disease	1.941 (1.121–3.361)	0.018	1.378 (0.419–4.530)	0.598
Serum AP level Tertile				
I (low)	1	–	1	–
II (middle)	1.529 (0.809–2.893)	0.191	1.857 (0.920–3.750)	0.084
III (high)	2.198 (1.197–4.036)	0.011	2.598 (1.331–5.071)	0.005
Antiplatelets	2.580 (0.930–7.153)	0.069		
Beta-blocker	0.770 (0.178–3.330)	0.726		
Calcium inhibitor	0.649 (0.349–1.207)	0.172		
ACEI	–	0.998		
Angiotensin II antagonist	0.712 (0.214–2.367)	0.580		
Diuretic	2.263 (0.472–10.851)	0.307		
Insulin	1.421 (0.537–3.760)	0.479		
Oral antidiabetic drug	2.137 (1.173–3.892)	0.013	1.779 (0.722–4.387)	0.211
Statin	2.736 (0.981–7.632)	0.055		
Atrial fibrillation	1.074 (0.317–3.640)	0.909		
Ventricular Tachycardia	2.59 (0.529–12.692)	0.240		
Heart arrest	3.026 (0.600–15.258)	0.180		
Pulmonary Infection	2.189 (0.928–5.164)	0.074		
Hydropericardium	1.795 (0.207–15.562)	0.596		

*TyG* triglyceride-glucose, *TG/HDL-C ratio* triglyceride/high-density lipoprotein cholesterol ratio, *STEMI* ST-elevation myocardial infarction, *WBC* white blood cell, *ACEI* angiotensin converting enzyme inhibitor, *Hb* hemoglobin, *CAD* coronary artery disease, *SBP* systolic blood pressure, *DBP* diastolic blood pressure, *RHR* resting heart rate, *LM* left main coronary artery, *LAD* left anterior descending artery, *LCX* left circumflex artery, *RCA* right coronary artery, *FBG* fasting blood glucose, *LDL-C* low-density lipoprotein cholesterol, *HDL-C* high-density lipoprotein cholesterol, *TG* triglycerides, *TC* total cholesterol, *UA* uric acid, *Lp(a)* lipoprotein (a), *ApoA1* apolipoprotein A1, *ApoB* apolipoprotein B, *BUN* blood urea nitrogen, *LVEF* left ventricular ejection fraction, *AP* alkaline phosphatase.

## Discussion

Our clinical study has, for the first time, reported that independent risk factors for AMI hospitalized patients with LVEF < 40% are serum AP levels, LAD as IRA, while an independent protective factor is TG. Insulin resistance surrogate markers did not show any significant association with the aforementioned factors. Furthermore, we found no significant correlation between serum AP and IR surrogate markers in AMI patients. However, a significant association still exists between previously established IR surrogate markers, TyG index, and TG/HDL-C ratio.

Serum alkaline phosphatase is a hydrolytic enzyme responsible for dephosphorylation processes in various types of molecules. It is expressed in multiple tissues, with the highest concentration found in bone, liver, and kidneys, and lower levels in the intestine, placenta, kidneys, and white blood cells^17^. In clinical practice, serum alkaline phosphatase is predominantly used for diagnosing obstructive biliary disease and monitoring metabolic bone diseases caused by renal insufficiency. Phosphate has been proven to be a protective factor for vascular integrity. During the dephosphorylation process mediated by AP, hydrolysis of phosphate may promote vascular calcification, ultimately leading to endothelial dysfunction^18^. Some novel inhibitors targeting the physiological pyrophosphatase activity of AP have demonstrated the ability to reduce vascular calcification in animal models^19^. Additionally, studies have shown an upregulation of serum AP levels in blood vessels with medial calcification, supporting the involvement of AP in vascular calcification mediation^20^. The promotion of vascular calcification by AP was initially discovered in maintenance hemodialysis patients, and higher levels of AP have been consistently associated with increased mortality rates in these patients^12^. As vascular calcification is a major contributor to atherosclerosis and leads to arterial stiffness, aging, and ultimately adverse cardiovascular events^21,22^, researchers have used intravascular ultrasound (IVUS) examinations on patients with acute coronary syndrome (ACS) to indicate AP as a potential predictive biomarker for calcification and plaque vulnerability^23^. Professor Wannamethee et al. not only found a strong correlation between AP and various cardiovascular risk factors but also conducted an 11-year follow-up study on a baseline population without CVD, revealing a significant association between elevated serum AP levels and increased risk of CAD events^24^. Another study investigating 470 stable angina patients found that higher serum AP levels were associated with higher Gensini scores and more severe forms of CAD^25^. In a study establishing a link between liver parameters and narrow diameters of coronary vessels in AMI patients, AP was identified as the most important variable for predicting coronary vessel diameter narrowing ≥ 50% or coronary vessel diameter < 50%^26^. Elevated serum AP during AMI, although below the upper limit of normal, still independently correlate with a higher risk of adverse cardiovascular and cerebrovascular events requiring primary percutaneous coronary intervention^27^. Moreover, research has found an independent relationship between AP and adverse outcomes in myocardial infarction survivors^28^. Research has also indicated that AP is a predictive factor for mortality, MI, or stent thrombosis after coronary artery drug-eluting stent implantation^13^. In recent years, studies have discovered that excessive tissue nonspecific alkaline phosphatase (TNAP) exacerbates myocardial fibrosis induced by MI^29^.

Insulin resistance is typically defined as a reduced sensitivity or responsiveness to the metabolic actions of insulin. Currently, the hyperinsulinemic-euglycemic clamp technique is considered the "gold standard" for assessing IR. However, the technique is time-consuming, labor-intensive, expensive, and requires experienced operators, making it impractical for epidemiological studies and large-scale clinical investigations^30^. In recent years, some new and simple indicators have been considered reliable surrogate markers for IR, such as the TyG index and TG/HDL ratio^31^. Numerous studies have found these surrogate markers to be independent risk factors for CVD and prognosis^32–34^. Recent research has also shown a positive and independent correlation between serum AP and surrogate markers of IR in the general population^11^. However, in our study population, we did not find a significant correlation between the two, but we did observe a significant correlation between the previously established surrogate markers of IR, TyG index, and TG/HDL-C ratio, which is consistent with previous research^4^.

Left ventricular function after AMI is the most important predictor of long-term prognosis^35^. Impaired LV function indicates a higher risk of cardiac arrest and death^36–38^. Some studies have indicated that higher TyG index is an independent and causal risk factor for heart failure in the general population^39^. However, we did not find a significant association between TyG index and severe LV dysfunction in patients hospitalized after AMI. Another study analyzed factors contributing to the decline in LVEF after PCI in patients with STEMI and found that infarction involving the LAD was a significant determinant of LVEF decline in young STEMI patients^40^, which is consistent with our results. It may be due to the LAD supplying 40% to 50% of the LV myocardium, and patients with LAD as IRA tend to experience more pronounced LV remodeling and dysfunction compared to those without LAD as IRA^41^. Elevated TG are often associated with CVD and adverse prognosis^42^.

Our study found that elevated TG were a protective factor for LVEF < 40% in patients hospitalized after AMI, but the specific mechanisms are unclear. The epidemiological studies from multiple previous cohorts consistently indicate a direct relationship between serum triglyceride levels and the risk of coronary heart disease^43–45^. Furthermore, a meta-analysis found that an increase in TG levels is associated with a dose-dependent elevation in the risk of cardiovascular disease and all-cause mortality^46^. In our study, triglycerides appear to act as a protective factor against heart failure following MI, although the specific mechanism remains unclear. We consider that this may be due to a decrease in triglyceride levels following myocardial infarction-induced heart failure. Several factors could contribute to this phenomenon: Firstly, evidence suggests a significant enhancement of non-cardiac lipolysis processes in heart failure patients^47^. Secondly, post-myocardial infarction, sympathetic nervous system activation stimulates the adrenergic system, which not only increases heart rate but also promotes the breakdown of non-cardiac fat tissues^48^. Thirdly, severe left ventricular dysfunction often accompanies insulin resistance, and damage to insulin signaling during heart failure can lead to non-cardiac fat breakdown^49^. Lastly, severe inadequacy in left ventricular contraction, leading to elevated B-type natriuretic peptide (BNP) levels, can further stimulate fat tissue lipolysis processes^50^.

Hypoxic liver injury caused by AMI may result in a slight elevation of serum AP, but studies suggest that it does not cause significant confounding of results^27^. However, the specific mechanism underlying the AMI remains unclear. We speculate that the elevation of serum AP levels may lead to: first, aggravated myocardial fibrosis, promoting ventricular remodeling^29,51^; second, endothelial dysfunction leading to poor microcirculation ^18^; third, inadequate collateral circulation of coronary arteries^52^; fourth, slow coronary blood flow^53^, ultimately resulting in severe impairment of LV function after AMI. Based on the first-time confirmation from our study, we have discovered an association between alkaline phosphatase and heart failure following myocardial infarction, prior to the administration of relevant treatments. Our research finding provides crucial evidence for the early prevention of left ventricular dysfunction after myocardial infarction. Furthermore, our research has concluded that alkaline phosphatase cannot serve as a substitute marker for insulin resistance in patients with myocardial infarction, as there is no significant correlation between the two.

### Study limitations

Our study had several limitations. Firstly, our study is retrospective in nature and cannot establish causal relationships between certain outcomes. Secondly, The sample size is relatively small, which may affect the reliability of the results. Thirdly, due to the lack of recorded insulin concentration data, we were unable to compare serum AP levels with HOMA-IR and the clamp test for hyperinsulinemia. Finally, our study population consisted exclusively of the Han Chinese ethnicity, thus making it difficult to generalize the current research findings to all countries and ethnic groups. Although adjustments were made for other potential risk factors, we cannot completely rule out the possibility of residual or unassessed confounding factors.

## Conclusion

Serum alkaline phosphatase and left anterior descending artery as infarct related artery are independent risk factors for severe reduction in LVEF during hospitalization for AMI. Conversely, triglyceride are independent protective factors for severe reduction in LVEF during AMI hospitalization. We also found a significant association between previous IR surrogate marker TyG index and TG/HDL-C ratio in AMI patients, but no significant correlation was observed between serum AP and IR surrogate markers.

## Data Availability

Due to the small amount of data in this study and the fact that we will collect further data for subsequent studies, the datasets generated and/or analysed during the current study are not publicly available but are available from the corresponding author on reasonable request.

## Abbreviations

AP: Alkaline phosphatase
AF: Atrial fibrillation
ACS: Acute coronary syndrome
TNAP: Tissue nonspecific alkaline phosphatase
LV: Left ventricular
LVEF: Left ventricular ejection fraction
PCI: Percutaneous coronary intervention
TyG: Triglyceride-glucose
IR: Insulin resistance
CAD: Coronary artery disease
CVD: Cardiovascular disease
TG: Triglyceride
SBP: Systolic blood pressure
DBP: Diastolic blood pressure
RHR: Resting heart rate
LM: Left main coronary artery
LAD: Left anterior descending artery
LCX: Left circumflex artery
RCA: Right coronary artery
IRA: Infarct related artery
FBG: Fasting blood glucose
LDL-C: Low-density lipoprotein cholesterol
HDL-C: High-density lipoprotein cholesterol
TG: Triglycerides
TC: Total cholesterol
UA: Uric Acid
Lp(a): Lipoprotein (a)
ApoA1: Apolipoprotein A1
ApoB: Apolipoprotein B
LVEF: Left ventricular ejection fraction
WBC: White blood cell
Hb: Hemoglobin
BUN: Blood urea nitrogen
MACCE: Main adverse cardiovascular and cerebrovascular events
BNP: B-type natriuretic peptide
PCI: Percutaneous coronary intervention
CABG: Coronary Artery Bypass Grafting
MI: Myocardial infarction
STEMI: ST-elevation myocardial infarction
NSTEMI: Non-ST-elevation myocardial infarction
NSTE-ACS: Non-ST elevation acute coronary syndrome
