# Author Correction: The relationship between insulin resistance, serum alkaline phosphatase, and left ventricular dysfunction following myocardial infarction

**DOI:** 10.1038/s41598-023-46685-w

**Published:** 2023-11-15

**Authors:** Qifeng Guo, Mengdan Miao, Linan Duan, Yongsheng Liu, Yahui Qiu, Xuejuan Feng, Shisen Liang, Weiqiang Xiao, Mingqi Zheng, Mei Wei, Gang Liu

**Affiliations:** 1 Department of Heart Center, the First Hospital of Hebei Medicical University, 89Donggang Road, Shijiazhuang, 050000 Hebei China; 2 Graduate School of Hebei Medical University, 361 Zhongshan East Road, Shijiazhuang, 050000 Hebei China; 3 Hebei Key Laboratory of Heart and Metabolism, Shijiazhuang, 050000 Hebei China; 4 Department of Geriatric Medicine, the First Hospital of Hebei Medicical University, 89 Donggang Road, Shijiazhuang, 050000 Hebei China

Correction to: *Scientific Reports* 10.1038/s41598-023-45246-5, published online 20 October 2023

In the original version of this Article, the authors Qifeng Guo and Mengdan Miao were omitted as equally contributing authors.

The original Article has been corrected.

